# Activation of telomerase activity and telomere elongation of host cells by *Theileria annulata* infection

**DOI:** 10.3389/fmicb.2023.1128433

**Published:** 2023-02-23

**Authors:** Junlong Liu, Shuaiyang Zhao, Zhi Li, Zhigang Zhang, Baocai Zhao, Guiquan Guan, Hong Yin, Jianxun Luo

**Affiliations:** ^1^State Key Laboratory of Veterinary Etiological Biology, Key Laboratory of Veterinary Parasitology of Gansu Province, Lanzhou Veterinary Research Institute, Chinese Academy of Agricultural Sciences, Lanzhou, Gansu, China; ^2^Qinghai Academy of Animal Sciences and Veterinary Medicine, Qinghai University, Xining, Qinghai, China; ^3^Jiangsu Co-Innovation Center for the Prevention and Control of Important Animal Infectious Disease and Zoonosis, Yangzhou University, Yangzhou, China

**Keywords:** *Theileria annulata*, host cell, telomere, telomerase, bTERT, bHSP90

## Abstract

*Theileria annulata*-transformed cells share many phenotypes with cancer cells, including uncontrolled proliferation, immortalization, and dissemination. Telomeres are DNA-protein complex at the end of eukaryotic chromosomes that function to maintain genome stability and cell replicative capacity. Telomere length maintenance is primarily dependent on telomerase activity. In up to 90% of human cancer cells, telomerase is reactivated through expression of its catalytic subunit TERT. However, the effect of *T. annulata* infection on telomere and telomerase activity in bovine cells has not yet been described. In the present study, we confirmed that telomere length and telomerase activity are upregulated after *T. annulata* infection in three types of cell lines. This change depends on the presence of parasites. After eliminating *Theileria* from cells with antitheilerial drug buparvaquone, telomerase activity and the expression level of bTERT were decreased. In addition, inhibition of bHSP90 by novobiocin led to decreased AKT phosphorylation levels and telomerase activity, indicating that the bHSP90-AKT complex is a potent factor modulates telomerase activity in *T. annulata*-infected cells.

## Introduction

*Theileria annulata* is a protozoan parasite transmitted by ticks of the genus *Hyalomma* and causes a tick-borne disease in cattle called tropical theileriosis. The disease is widely distributed ranging from the parts of Southern Europe and Northern Africa to the Near and Middle East and to India and China in Asia (Unlu et al., 2018), and causes important economic losses in the productivity of cattle. At the initial infection stage, sporozoites invade host leucocytes and develop into schizonts. The schizont-infected cells obtained ability of uncontrolled proliferation and lead to the enlargement of lymph nodes. *T. annulata* is able to infect and transform lymph cells, including monocytes/macrophages, B cells, and dendritic cells (Spooner et al., 1989). To date, the genus *Theileria*, including *T. annulata, T. parva,* and *T. lestoquardi,* is the only eukaryote that has been found to transform host cells. Following sporozoite invasion, the schizont stage develops in the cytoplasm of host cells and leading host cell to uncontrolled proliferation without any exogenous stimulus. Several host cell signaling pathways including JNK, NF-κb, and c-Myc are activated due to *Theileria* infection (Heussler et al., 1999; Dessauge et al., 2005; Lizundia et al., 2006; Marsolier et al., 2015). However, the transformation induced by *Theileria* is reversible by parasite elimination with the antitheilerial drug BW720c.

Telomeres are at the end of chromosomes and play a key role in avoiding DNA damage during progressive cell division. In normal cells, telomeres are shortened by l 50–200 base pairs for each cell division (Harley et al., 1990). When the length of telomeres reaches a critical level, the DNA damage signal will be initiated, leading to cell cycle and replicative arrest (Campisi, 1997). Therefore, maintaining the length of telomeres is important to keep the replicative captivity of some cell types, such as stem cells or immortalized cells. The canonical telomere length maintenance occurs through telomerase to synthesize telomeric repeats at the end of telomere (Nguyen and Wong, 2020). Telomerase is a ribonucleoprotein complex that compromises a catalytic protein with telomere-specific reverse transcriptase (TERT) activity and internal RNA template (TERC) (Celeghin et al., 2016). Studies of the cells that express telomerase enzyme indicated that the transcription of TERT is the key factor in the regulation of telomerase activity (Roake and Artandi, 2020). TERT is usually silenced in normal somatic cells; however, its expression lever is significantly upregulated in more than 90% of human cancer cells (Jafri et al., 2016). The mechanisms to maintain telomere length by telomerase in cancer cells include mutation of the TERT promoter, abnormal amplification of the TERT and TERC genes, rearrangements of the TERT gene, and genetic variants of the TERT gene and its promoter (Gaspar et al., 2018). The promoter of the human TERT gene contains several regulatory elements that many reported factors can interact directly and regulate the expression of TERT. A previous study confirmed that c-Myc could bind to the E-box of the promoter of hTERT and upregulate the expression of the gene and the activity of telomerase (Wu et al., 1999).

Except for endogenous factors that have the ability to activate telomerase and thus to maintain telomere length, many oncoviruses have been confirmed to activate telomerase of host cells through various strategies. The human papillomavirus E6 oncoprotein could increase cellular telomerase activity by upregulating of the human TERT (Klingelhutz et al., 1996; Veldman et al., 2001). Another study reported that the E6 protein interacts directly with hTERT and modulates telomerase activation (Liu et al., 2009). Epstein–Barr virus latent membrane protein 1 can activate the hTERT promoter of B cells and enhance telomerase activity (Terrin et al., 2008). *Theileria-*transformed cells share many characteristics with cancer cells, including immortalization, uncontrolled proliferation and dissemination (Tretina et al., 2015). However, the effect of *Theileria* infection on host cell telomerase activity has not yet been reported. A previously study showed that the telomerase inhibitor MST-312 could effectively restrict the proliferation of *T. parva*-infected cells (Hayashida et al., 2013). However, the expression level of bTERT in bovine B-lymphosarcoma cells was not changed during *T. annulata* infection (Durrani et al., 2012; Kinnaird et al., 2013).

A recent study reported that the binding of telomerase to the NF-κB p65 subunit could enhance the expression level of some genes, including IL-6 and TNF-α, suggesting that the binding is able to maintain telomerase activity in cancers (Ghosh et al., 2012). In *Theileria*-infected cells, many studies have confirmed that the host cells NF-κB signaling pathway is activated during the infection (Palmer et al., 1997; Schmuckli-Maurer et al., 2010). Thus, we hypothesized that *Theileria* infection could activate the host cells telomerase and elongate telomere length. In this study, we analyzed telomere length, the mRNA and protein expression levels of bTERT, and the telomerase activity of three *T. annulata*-infected cell lines to elucidate the relationship of telomerase activity with the intracellular infection of *T. annulata*.

## Materials and methods

### Cell culture

TaNM1 is a bovine lymphocyte cell line derived from a cow infected with Inner Mongolia isolate of *T. annulata*. TaBC is a bovine B cell line infected with the *T. annulata* Kashi isolate (Rashid et al., 2019). TaDC cell line is a bovine dendritic cell line infected with the *T. annulata* Kashi isolated *in vitro* (Liu et al., 2020). The transformed cells were cultured in RPMI 1640 (Biological Industries, Israel) supplemented with 10% fetal bovine serum (Biological Industries, Israel). HeLa cells were cultured in DMEM medium (Gibco) containing 10% fetal bovine serum (Biological Industries, Israel). All cells were cultured at 37°C in a humidified 5% CO_2_ incubator. Normal bovine peripheral blood mononuclear cells (PBMCs), B cells, and dendritic cells were isolated as previously described (Rashid et al., 2019; Liu et al., 2020), the isolated cells were stored in liquid nitrogen until use.

### Treatment of cells with different reagents

*Theileria annulata*-infected cells TaNM1 were cultured in 12-well culture plates at 10^5^ cells per well and treated with 200 ng/ml (stock 2 mg/ml in ethanol) buparvaquone (BW720c, Sigma). Meanwhile, cells were treated with the same amount of ethanol that used for dissolve BW720c as the control group. HeLa cells were also treated with the same concentration of BW720c to test its effect on the protein level of telomerase reverse transcriptase.

5 × 10^5^ TaNM1 cells were treated with 30 nmol/ml BIBR, 300 nmol/ml novobiocin, and 1 nmol/ml Geldanamycin (MedChemExpress), respectively. The equal volume of DMSO was used to treat the cells as control group.

### DNA extraction

DNA of the cell samples was extracted by using the DNeasy Blood and Tissue spin column protocol (Qiagen) following the instruction manual. The concentration of the extracted DNA was measured by NanoDrop-2000 Spectrophotometer (NanoDrop Technologies, Wilmington, DE, United States), and the DNA was diluted to a final concentration of 20 ng/μl. In addition, the integrity of each DNA sample was tested as previously described method (Seeker et al., 2016).

### Relative telomere length determination

The relative telomere length (RTL) was measured based on the published qPCR method (Cawthon, 2002). The telomere primers were used as published (Cawthon, 2009), and the sequence of the primers was TELG, 5’-ACACTAAGGTTTGGGTTTGGGTTTGGGTTTGGGTTAGTGT-3′, and TELC, 5’-TGTTAGGTATCCCTATCCCTATCCCTATCCCTATCCCTACA-3′. The single-copy gene ZAR1 was used as a reference gene, and the sequence for the forward and reverse primers were 5’-AAGTGCCTATGTGTGGTGTG-3′ and 5’-CAGGTGATATCCTCCACTCG-3′. The final volume of PCR reaction system contained 10 μl 2 × TB Green Premix Ex Taq (TaKaRa), 1 μM of each primer, 1 μl DNA, and DNase-free water to a final volume of 20 μl. Real-time quantitative PCR was carried out in a Stratagene Mx3005P instrument. The reaction condition for telomere gene was as follows: 1 cycle of 15 min at 95°C, 2 cycles of 15 s at 94°C, 15 s at 49°C, 45 cycles of 94°C for 15 s, 62°C for 10 s, and 15 s at 74°C for signal acquisition (Cawthon, 2009). The thermal cycler profile for the reference gene was 1 cycle of 3 min at 98°C, 45 cycles of 10 s at 95°C, and 60 s at 60°C for signal acquisition. The RTL was determined by the ratio of the telomere product amplification (T) versus that of the reference gene (S) (Cawthon, 2002; Gilchrist et al., 2015). All samples were tested three times.

### Quantitative PCR analysis of gene transcriptional levels

Total RNA was extracted from the cell samples by RNeasy Mini Kit (Qiagen, Germany), and the concentration of each sample was determined by using NanoDrop 2000 (NanoDrop Technologies, Wilmington, DE, United States). One microgram of RNA was used for cDNA synthesis using the PrimeScript™ RT Reagent Kit with gDNA Eraser (TaKaRa). The qPCR reactions were performed with TB Green Premix Ex Taq (TaKaRa) using Stratagene Mx3005P (Agilent Technologies). The reaction system was 95°C 30 s, 40 cycles of 95°C 20 s, 60°C 30 s, 1 cycle of 95°C 15 s, 60°C 1 min, and 95°C 30 s. For *T. annulata* genes detection, the reaction system was 95°C 3 min; 40 cycles of 95°C 30 s, 60°C 1 min, 1 cycle of 95°C 1 min, 58°C 30 s, and 95°C 30 s. The information of primers used for quantitative PCR is shown in Table 1.

**Table 1:** Primers used for qPCR in the present study.

Name	Sequences	Size (bp)	Reference
b-TERT-F	5′-CAGCAGCCTCTTCAACCTCTTC-3′	149	Present study
b-TERT-R	5′-TGTTCTCCATGTCCCCATAGCAG-3′		
b-TERC-F	5′-TACCGCCATCCACCATCCAG-3′	210	Garrels et al. (2012)
b-TERC-R	5′-TCTTCACGGCGGCAATGGAC-3′		
b-HSP90-F	5′-GAGAGCTTGACCGATCCCAG-3′	96	Tang et al. (2021)
b-HSP90-R	5′-GTCCACGATGGTGAGGGTTC-3′		
b-B2M-F	5′-CGGGGAGAAGATTAAATCGGAGCAG-3′	75	Present study
b-B2M-R	5′-GCGTGGGACAGAAGGTAGAAAGAC-3′		
h-TERT-F	5′-GCCGATTGTGAACATGGACTACG-3′	113	Huang et al. (2015)
h-TERC-R	5′-GCTCGTAGTTGAGCACGCTGAA-3′		
h-actin-F	5′-GTGAAGGTGACAGCAGTCGGTT-3′	157	
h-actin-R	5′-GAAGTGGGGTGGCTTTTAGGA-3′		
TaSP-F	5′-AGCAGCCCCTTGTCATGGG-3′	284	Hostettler et al. (2014)
TaSP-R	5′-TAATAGCTTTTGCACGGAGGA-3′		
Ta-actin-F	5′- GAGACCACCTACAACAGCATCATG −3′	182	
Ta-actin-R	5′- CACCTTGATCTTCATGGTGCTGGG −3′		
Ta-TERT-F	5′-ATACAGACTTTCACGCTTTACG-3′	174	Present study
Ta-TERT-R	5′-AATCTTCTCCAGGTCGCACA-3′		

b, bovine; h, human; Ta, *Theileria annulata*.

### Preparation of cell lysate

The target cell samples were washed with phosphate-buffered saline (PBS) two times, and the cell number was counted using Countstar cell analysis system (Alit Biotech, Shanghai, China). A total of 1 × 10^6^ cells were re-suspended in 200 μl CHAPS lysis buffer (Merck-Millipore) and incubated on ice for 30 min. The cell lysate was then centrifuged at 12,000 ×*g* for 20 min at 4°C. The protein concentration was determined by using Pierce BCA protein assay kit (Thermo Fisher Scientific). The supernatant were then transferred into new sterile tubes and stored at −80°C.

### Telomerase activity test

The activity of telomerase was tested based on the classical TRAP method (Kim and Wu, 1997) with minor modification. Briefly, cultured cells were washed with PBS and the cell numbers were counted. A total of 1 × 10^6^ cells were lysed with CHAPS lysis buffer for 30 min on ice. After centrifugation at 12,000 ×*g* for 20 min, the supernatant was removed into a new prechilled tubes and the concentration was determined by the Pierce BCA Protein Assay Kit (Thermo Scientific). Two steps were applied for TRAP assay. The first reaction system contained 1 μl cell lysate, 1 μl dNTP (2.5 mM), 1 μmol/l TS primer (5‘-AATCCGTCGAGCAGAGTT-3’), 1 μl 10 × TRAP buffer (200 mM Tris–HCL, 15 mM MgCL_2_, 630 mM KCL, 0.5% Tween 20, 10 mM EGTA, and 0.1% BSA, pH 8.3), and 6.5 μl ddH_2_O. The mixture were incubated at 30°C for 40 min, inactivated at 95°C 5 min, and stored at 4°C. The second step was PCR reaction, each 20 μl reaction system contained 2 μl of products from the first step, 2 μl 10 × TRAP buffer, 3 μl dNTP (2.5 mM), 0.5 μmol/l TS primer, 0.5 μmol/l ACX primer (5‘-GCGCGGCTTACCCTTACCCTTACCCTAACC-3’), 0.5 μmol/l NT primer (5‘-ATCGCTTCTCGGCCTTTT-3′), 0.5 amol/μl TSNT (5‘-AATCCGTCGAGCAGAGTTAAAAGGCCGAGAAGCGAT-3′), and 11 μl ddH_2_O. PCR was performed at 95°C for 3 min, 30 cycles of 94°C for 30 s, 59°C for 30 s, 72°C for 1 min, and 72°C for 5 min. PCR products were separated on a 15% native-PAGE gel in TBE buffer. After electrophoresis, the gel was stained with GelRed for 30 min and the images were captured under UV light with reverse processing.

### Western blotting analysis

The cell lysate was mixed with 5× loading buffer and heated at 95°C for 10 min. The proteins were then separated by 12% SDS-PAGE and transferred onto a nitrocellulose membrane (0.2 μM, Whatman) for 2 h at 200 mA. Membrane was then blocked with 3% bovine serum albumin in TBST buffer for 1 h at 4°C. Primary antibodies were diluted in 1% BSA/TBST according to the manufacture’s instruction and incubated with the membrane overnight at 4°C. After washed with TBST buffer, horseradish peroxidase (HRP)-conjugated secondary antibody was then incubated with the membrane for 1 h at 4°C. After washed with TBST buffer, the HRP-conjugated secondary antibody was performed by SuperSignal West Atto Ultimate Sensitivity Chemiluminescent Substrate (Thermo Scientific). Information on the antibodies used in the present study is shown in Table 2.

**Table 2:** Information for primary antibodies used in the present study.

Antibody	Host species	Molecular weight	Dilution rate	Company
TERT	Rabbit	127 kDa	1:1000	Abcam
β-actin	Mouse	42 kDa	1:5000	Abcam
Akt	Rabbit	59 kDa	1:2000	Abcam
Phospho-Akt (Ser473)	Rabbit	59 kDa	1:2000	Cell Signaling Technology
HSP90	Rabbit	90 kDa	1:4000	Proteintech

### Statistical analysis

The experimental results were statistically analyzed by using GraphPad PRISM 8.0 software (GraphPad Software Inc., San Diego, CA, United States). The results presented in all the figures represent the mean of three independent experiments. Student’s *t*-test or one-way ANOVA with multiple comparisons test was applied for statistical analysis. *p* < 0.05 were considered statistically significant.

## Results

### Telomeres of host cells elongated after *Theileria annulata* infection

**Figure 1:**
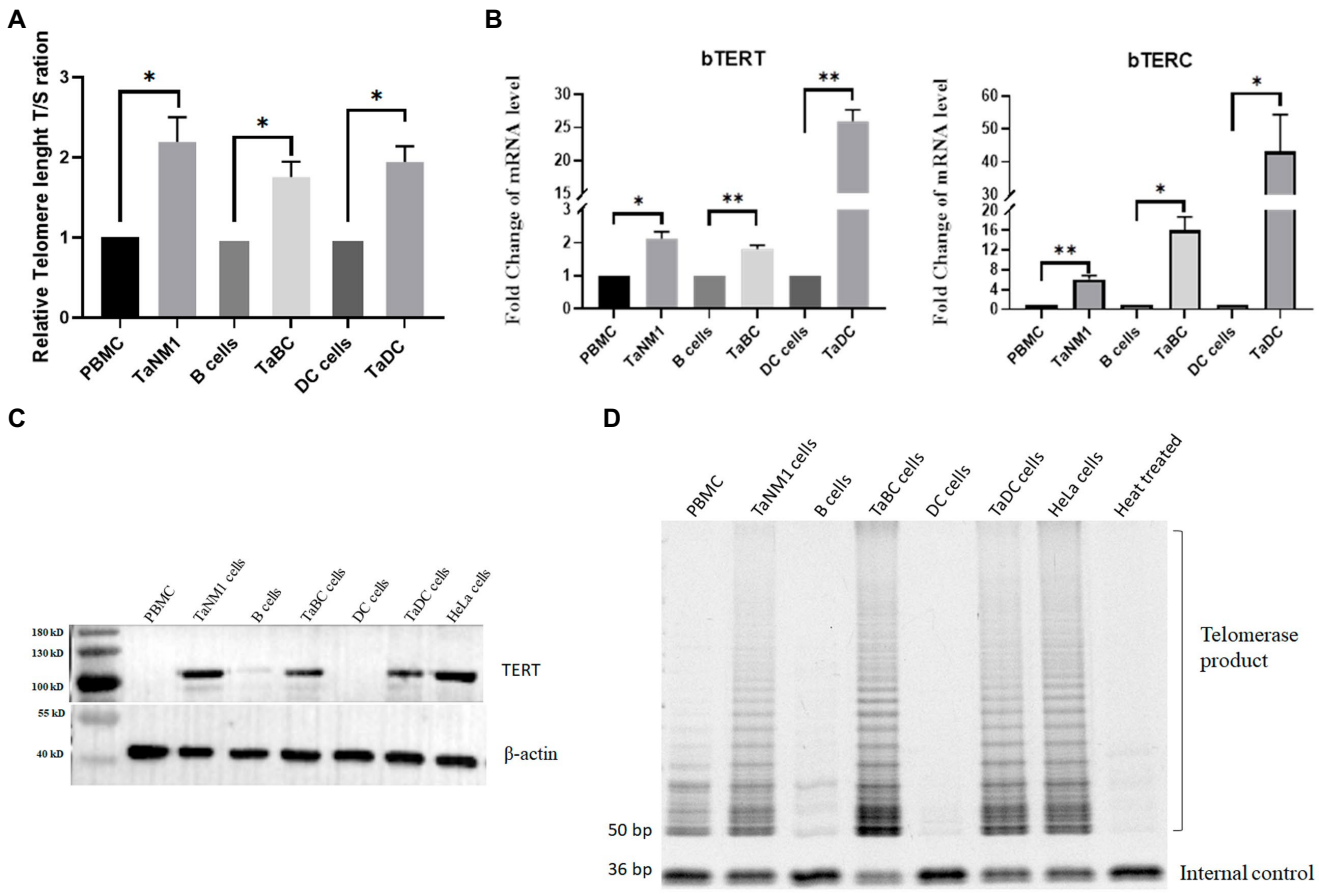
*Theileria annulata* infection increased telomere length and telomerase activity. **(A)** The relative telomere length is significantly increased after *T. annulata* infection in three types of cell lines. Normal PBMCs, B cells, and DCs cells were used as control group. **(B)** Transcriptional analysis of the bTERT and bTERC genes in normal cells and *T. annulata*-infected PBMCs, B cells DCs. **(C)** Western blot analysis of the protein expression level of bTERT in the cells before and after *T. annulata* infection. HeLa cell lysates were taken as the TERT-positive group. **(D)** TRAP assay analysis of the telomerase activity of normal cells (PBMCs, B cells, and DCs) and *T. annulata*-infected cells (TaNM1, TaBC, and TaDC). HeLa cell lysates were taken as telomerase-positive control. Heat-treated (100°C for 10 min) TaNM1 cell lysates were taken as telomerase-negative control. The Student’s T test was used for statistical analysis from the results of three independent experiments. The error bars denote the standard error. *p < 0.05, **p < 0.01.

Aim to investigate the change of the telomere length of host cells after *Theileria* infection, we analyzed the telomere length of normal bovine cells including PBMCs, B cells, DC cells and their counterpart *T. annulata*-infected cell lines. A significant increase of the RTL was found in all three cell lines compared to normal cells (Figure 1A). This result indicated that *T. annulata* infection could elongate the telomere length to avoid DNA damage response triggered cells apoptosis.

### bTERT and bTERC expression and telomerase activity in *Theileria annulata*-infected cells

Telomerase activation is a key factor in maintaining the telomerase length of cells. The expression of TERT and TERC is associated with the activity of telomerase. Thus, we examined the transcription levels of bTERT and bTERC in *T. annulata*-infected cells and compared them with those in normal cells. The results showed that the mRNA levels of the bTERT and bTERC gene of host cells was significantly upregulated after *T. annulata* infection in all three cell types (Figure 1B). Next, the protein level of bTERT was detected in both normal cells and *T. annulata*-infected cells. HeLa cells were used as TERT-positive samples. From the western blot results, bTERT protein was highly expressed after *T. annulata* infection, while bTERT protein expression was not found in normal cells (Figure 1C).

Subsequently, protein extracts from the different types of cell lysates were used to test the telomerase activity. HeLa cells and heat-treated TaNM1 cell lysate were used as positive and negative controls, respectively. The results showed that telomerase activity was found in all three types of *T. annulata*-infected cells. While in normal cells, the telomerase activity was either not present or much weaker than that in *T. annulata*-transformed cells (Figure 1D).

### BIBR restricts *Theileria annulata*-transformed cells proliferation but has no effect on parasites

**Figure 2:**
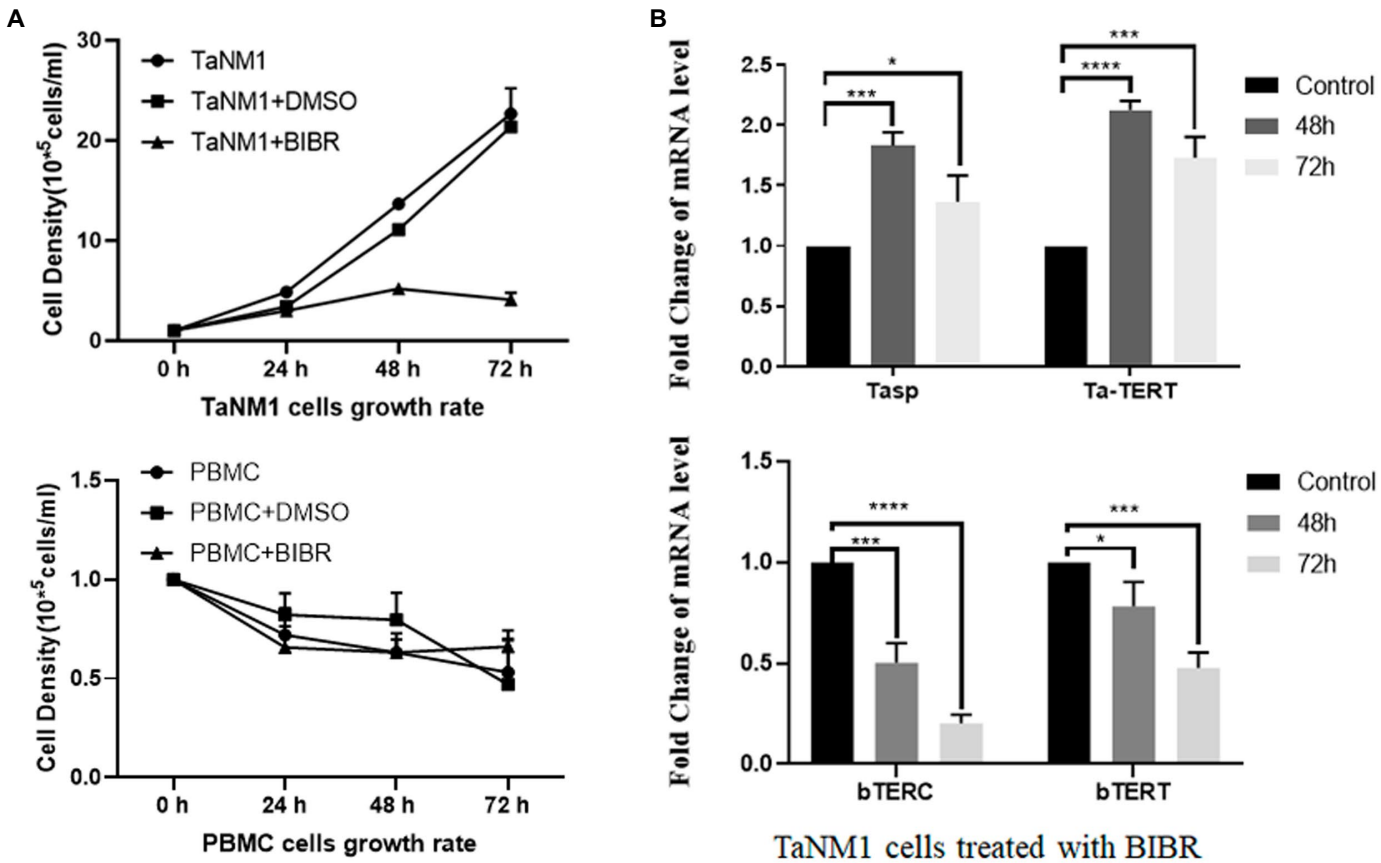
Effect of the telomerase inhibitor BIBR on TaNM1 cells: **(A)** BIBR at a concentration 30 nmol/ml inhibit the proliferation of *T. annulata*-infected cells, while the dissolve reagent DMSO group had no effect on cell growth compared with the blank control. In PBMC groups, the BIBR has no significant effects on cells proliferation compared with the blank and DMSO control group. Cell numbers were counted daily from three replicate cultures for each condition. **(B)** Transcriptional analysis of host genes and parasite genes after BIBR treatment. The transcription levels of host cell bTERT and bTERC genes were significantly downregulated after treatment. Transcription of Tasp and Ta-TERT of *T. annulata* was not restricted by BIBR. The results are the average of three independent experiments. The one-way ANOVA was used for statistical analysis. *p < 0.05, ***p < 0.001, ****p < 0.0001.

Telomerase plays an important role in cellular survival, and inhibition of telomerase activity induces programmed cell death. To test the contribution of telomerase activity on the proliferation of *T. annulata*-transformed cells, we treated TaNM1 cells with the telomerase-specific inhibitor BIBR for 24, 48, and 72 h. The concentration of the cells was measured and the results showed that *T. annulata* induced cell growth was arrested by BIBR (Figure 2A). From the BIBR-treated cell samples, the transcription levels of bTERT and bTERC were found to decrease significantly after treatment. In contrast, the transcription levels of parasite genes Tasp and TERT were significantly upregulated after BIBR treatment (Figure 2B).

### Buparvaquone treatment suppress the expression of bTERT and bTERC and telomerase activity of *Theileria annulata*-infected cells

**Figure 3:**
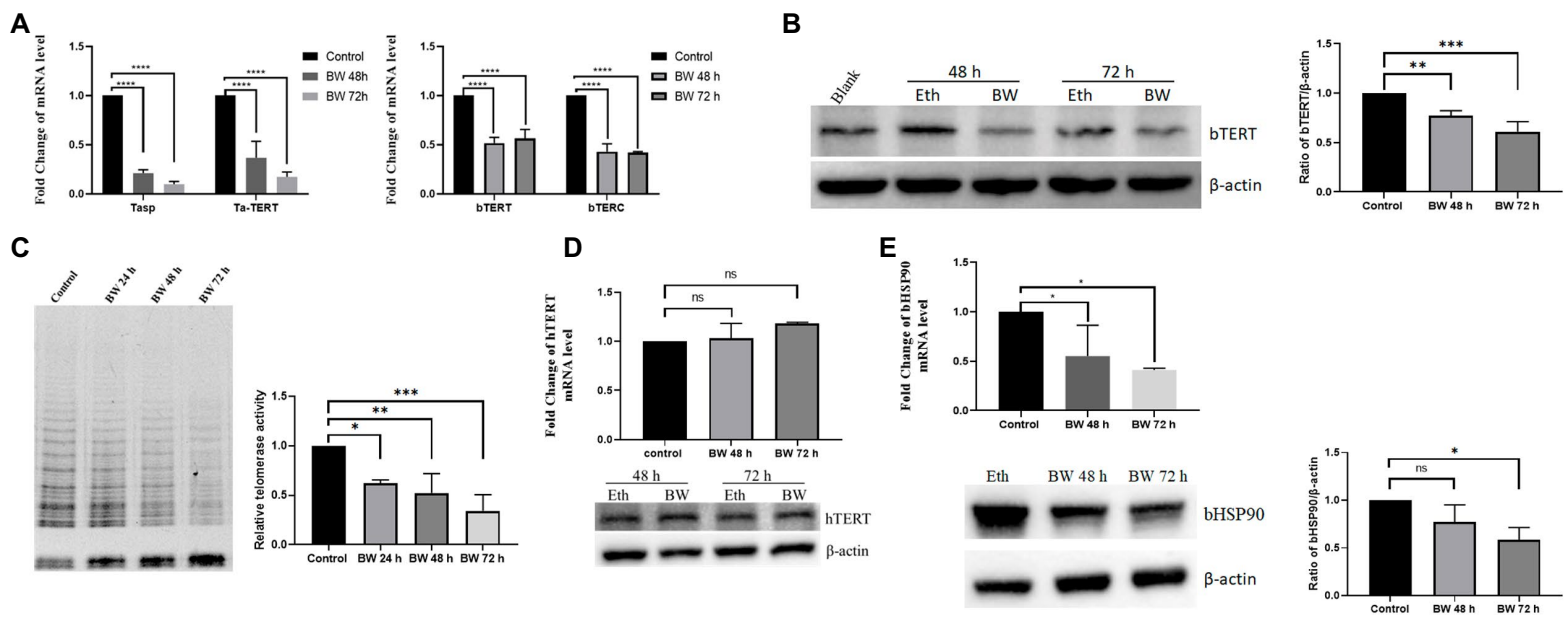
Effect of buparvaquone (BW) on the telomerase activity of host cells: **(A)** Changes in the transcription levels of *T. annulata* genes (Tasp., Ta-TERT) and host cell genes (bTERT, bTERC) after treatment with 200 ng/ml BW. **(B)** Western blot analysis of bTERT expression after cells treated with BW (left). The blank group is the normal cells. Eth means the cells incubated with a same amount of ethanol as in BW group. Quantification of Western blot for bTERT protein levels using ImageJ software (right). **(C)** Telomerase activity detection by TRAP assay. The control group consisted of cells cultured with ethanol (left). Quantification of relative telomerase activity by using ImageJ software (right). **(D)** HeLa cells were treated with 200 ng/ml of BW for 48 and 72 h and then, the hTERT transcription and expression levels were analyzed by Q-PCR and Western blot, respectively. **(E)** The expression level of bHSP90 was decreased after parasites were eliminated by BW from TaNM1 cells. Quantification of Western blot for bHSP90 protein levels using ImageJ software. The one-way ANOVA was used for statistical analysis (*n* = 3 times independent experiments). ns means no significant difference, *p < 0.05, **p < 0.01, ***p < 0.001, ****p < 0.0001.

Next, we aimed to confirm that the expression of bTERT and bTERC genes is induced by the infection of *T. annulata*. TaNM1 cells were treated with antitheilerial drug buparvaquone to eliminate parasites from the host cells. From the qPCR results, the transcription levels of *T. annulata* Tasp and taTERT genes were significantly downregulated after 48 h and 72 h treatment, which confirmed that the drug was effective. Following the elimination of *T. annulata*, bTERT and bTERC were found downregulated at the transcription level (Figure 3A). The protein level of bTERT was also lower than that in the control group according to the Western Blot results (Figure 3B). Cells lysate samples collected at 24 h, 48 h, and 72 h after BW treatment were used to test telomerase activity with the TRAP assay. The results showed that the amplified bands from the specific primer on the gel decreased gradually following the time of drug treatment (Figure 3C). To confirm that BW has no effect on the expression level of TERT, HeLa cells were incubated with the same concentration of BW. After treatment, both the protein and transcription levels of hTERT and hTERC were not significantly changed (Figure 3D). The results here confirmed that *T. annulata* infection is the main factor inducing the expression of bTERT and bTERC of its host cells, and to activating telomerase to maintain telomere length. In addition, we found that bHSP90 was also downregulated in both transcriptional and protein levels (Figure 3E), which indicated that bHSP90 might be a potential factor on telomerase activity in *T. annulata-*transformed cells.

### Inhibition of bHSP90 reduced Akt phosphorylation and telomerase activity in *Theileria annulata*-transformed cells

**Figure 4:**
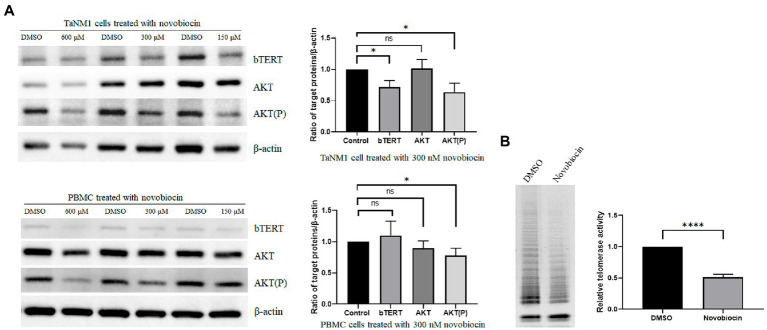
Effects of novobiocin on the expression of bTERT and telomerase activity: **(A)** The TaNM1 and PBMC cells were treated with different concentrations of novobiocin for 18 h. Western blot analysis of protein expression (bTERT, AKT(P), AKT) in TaNM1 and PBMC cells (left). Quantification of Western blot for the target proteins from the cells treated with 300 nM novobiocin using ImageJ software (right). **(B)** Telomerase activity detection after cells treated with 300 nM novobiocin for 18 h by using TRAP assay (left). Quantification of relative telomerase activity by using ImageJ software (right). The Student’s *T*-test was used for statistical analysis from the results of three independent experiments. The error bars denote the standard error. ns means no significant difference, *p < 0.05, ****p < 0.0001.

In human cells, the hHSP90 protein was confirmed participated in regulation of telomerase activity (Haendeler et al., 2003). The aim was to investigate the role of bHSP90 on telomerase activity in *T. annulata*-transformed cells. The cells were incubated with novobiocin for 18 h. From the western blot results, the protein levels of bTERT and phosphorylated AKT were reduced at concentrations of 300 μM and 150 μM novobiocin (Figure 4A). Telomerase activity was also found to be reduced by novobiocin at 300 μM (Figure 4B). The results of this part indicated that bHSP90 plays a similar role as hHSP90 in cancer cells on telomerase activity in *T. annulata*-transformed cells.

## Discussion

Our observations confirmed that *T. annulata* infection reactivated telomerase activity by increasing the expression level of the catalytic subunit of telomerase bTERT for the first time. The expression of bTERT is dependent on the presence of *T. annulata*. Once parasites were eliminated by the antitheilerial drug buparvaquone, the expression of bTERT and telomerase activity of host cells was reduced. We demonstrated that in all three types of *T. annulata*-transformed cells, the relative length of telomeres was significantly higher than that in the original cells. This might be an important factor in maintaining the immortalized phenotype of *T. annulata*-infected cells.

Telomerase activity is closely associated with its catalytic core which is composed of TERC and TERT (Schmidt and Cech, 2015). The expression of hTERC exists in most cells, but hTERT expression is absent in adult somatic cells where telomerase is inactivated. Thus, the expression level of TERT is considered the rate-limiting determinant of telomerase activity in telomerase-positive tumors (Yuan et al., 2019). In the present study, we demonstrated that bTERT expression level and telomerase activity is related to the presence of *T. annulata* in its transformed cells. The results here are contrast with a previous report that indicated the bTERT expression level was not changed in *T. annulata*-infected bovine B-lymphosarcoma cells (Durrani et al., 2012; Kinnaird et al., 2013). The reason for this difference might be due to the different original cell types. In our study, the cell lines were derived from normal bovine PBMCs, B cells, and DCs, which are telomerase-negative cells. While B-lymphosarcoma cells are tumor cells that express TERT due to chromosomal translocations (Nagel et al., 2010). We hypothesized the enhancement of bTERT expression by *T. annulata* in TERT-negative cells plays a key role to maintain cells immortalization, but this effect is not significant in TERT-positive cells. And a review report was also proposed that *T. annulata* used an alternative mechanism to maintain telomere length, or that already existed telomerase was hyperactivated in B-lymphosarcoma cells (Tretina et al., 2015). However, no studies have shown whether *T. annulata-*infected B-lymphosarcoma cells turn to death when telomerase activity is inhibited. However, one study showed that telomerase inhibitor MST-312 effectively inhibited proliferation of *T. parva*-infected T cells (Hayashida et al., 2013, 2). In our study, another telomerase-specific inhibitor BIBR was used. Similar to *T. parva*-transformed cells, the proliferation of *T. annulata*-infected cells was arrested. However, the transcriptional levels of the *T. annulata* genes Tasp and taTERT were not affected by BIBR treatment. Thus, we speculate that telomerase activity plays a key role in *T. annulata*-transformed cells derived from telomerase-negative cells.

In human tumor cells, many endogenous proteins were confirmed interact with TERT and enhance telomerase activity. One of those proteins is hHSP90. The complex of hHSP90 with its client proteins p23 and FKBP52 was reported to help hTERT move into the nucleus in lung cancer cells (Holt et al., 1999; Jeong et al., 2016). While the Akt-hHSP90-hTERT protein complex was demonstrated conducive to phosphorylate hTERT and thus increased telomerase activity (Haendeler et al., 2003). Inhibition of the interaction of AKT and hHSP90 by novobiocin results in dephosphorylation of Akt and inhibit telomerase activity. In addition, the phosphorylation of serine 227 of the bipartite nuclear localization signal sequence mediated by AKT contributes to the nuclear import of hTERT (Chung et al., 2012). In the present study, treatment of *T. annulata*-transformed bovine cells with novobiocin decreased the phosphorylation of AKT (Ser473) and downregulated telomerase activity. Together with a previous report that confirmed that AKT is constitutively activated in both *T. parva*- and *T. annulata*-infected cells (Heussler et al., 2001), we proposed that AKT regulates telomerase activity through the complex AKT -bHSP90-bTERT complex in *T. annulata*-transformed cells.

Recent work has shown that the protein complex of hTERT and the subunit of NF-κB p65 could bind to a subset of NF-κB and transcriptionally increase some gene levels including hTERT (Ghosh et al., 2012). In both *T. parva- and T. annulata*-transformed cells, NF-κB was found to be constitutively activated by the presence of parasites (Schmuckli-Maurer et al., 2010). Thus, the *Theileria*-dependent activation of NF-κB was speculated to contribute to the expression of bovine telomerase (Tretina et al., 2015). However, in our study, when *T. annulata*-infected cells were treated with the NF-κB inhibitor QNZ (EVP4593), the proliferation of the cells and bTERT transcription level was not found inhibited (data not shown). In human cells, c-Myc is known to induce hTERT expression by directly binding to the promoter of hTERT and enhancing telomerase activity (Wu et al., 1999). The expression of c-Myc was demonstrated to be upregulated through activation of the JAK2/STAT3 signaling pathway, which led to the transcription of c-Myc (Dessauge et al., 2005). Whether or not highly expressed c-Myc contributes to telomerase activity in *Theileria*-transformed cells needs to be confirm.

The genome of *T. annulata* is 8.35 Mb which contains approximately 3,792 protein-coding genes, among those genes, 689 proteins were identified with a predicted signal peptide (Gardner et al., 2005; Marsolier et al., 2015). However, the identified proteins accounted for small part of the predicted *T. annulata* proteins. Most of the studied proteins are localized on the schizont surface, and only a few proteins including TaPIN1, GcPE, Ta9, SuAT1, and TashHN are distributed in the cytoplasm or nucleus of the host cells (Tajeri and Langsley, 2020). None of the identified *Theileria* proteins were related to telomerase activity. Therefore, the parasite proteins that contribute to host cell telomerase activity and in which way they used need to be explored in future works.

## Conclusion

The present study confirmed for the first time that *T. annulata* infection reactivates telomerase activity and elongates telomere length of host cells. Our data clearly show that the expression of bTERT and telomerase is *Theileria* dependent. Once the parasite is eliminated by the antitheilerial drug buparvaquone, the expression of bTERT and telomerase activity was decreased and lead to cell death. Because the expression level of bHSP90 was reduced following the elimination of *T. annulata*, and the application of novobiocin restricted telomerase activity. We speculated that bHSP90 participates in the regulation of telomerase activity during *T. annulata* infection. How does *T. annulata* alter TERT expression? Are there parasite proteins involved for telomerase adjustment? Further studies are needed to answer these questions.

## Data availability statement

The original contributions presented in the study are included in the article/supplementary material, further inquiries can be directed to the corresponding authors.

## Author contributions

JLiu designed the experiment and wrote the draft of the manuscript. ZL and SZ did western blot works. BZ and ZZ did qPCR works. GG, HY, and JLuo corrected the manuscript. All authors contributed to the article and approved the submitted version.

## Funding

This study was financially supported by NSFC (no. 31972706). The Agricultural Science and Technology Innovation Program (CAAS-ASTIP-2016-LVRI). NBCITS (CARS-37). The Science Fund for Creative Research Groups of Gansu Province (22JR5RA024). The Leading Found of Lanzhou Veterinary Research Institute, CAAS (LVRI-SZJJ-202105). The hatching program of SKLVEB (SKLVEB2021CGQD02).

## Conflict of interest

The authors declare that the research was conducted in the absence of any commercial or financial relationships that could be construed as a potential conflict of interest.

## Publisher’s note

All claims expressed in this article are solely those of the authors and do not necessarily represent those of their affiliated organizations, or those of the publisher, the editors and the reviewers. Any product that may be evaluated in this article, or claim that may be made by its manufacturer, is not guaranteed or endorsed by the publisher.
